# Supplementary material for: Structural basis for bacterial energy extraction from atmospheric hydrogen
Source: Nature. 2023 Mar 8;615(7952):541–7. doi: 10.1038/s41586-023-05781-7 (PMC10017518; doi:10.1038/s41586-023-05781-7)
Supplement: Supplementary file 1 — This file contains Supplementary Figs. 1–5; Supplementary Tables 1–4; Supplementary Notes 1–4 and references. [file 41586_2023_5781_MOESM1_ESM.pdf]

nature portfolio

https://doi.org/10.1038/s41586-023-05781-7

Supplementary information

Structural basis for bacterial energy extraction from atmospheric hydrogen

In the format provided by the authors and unedited

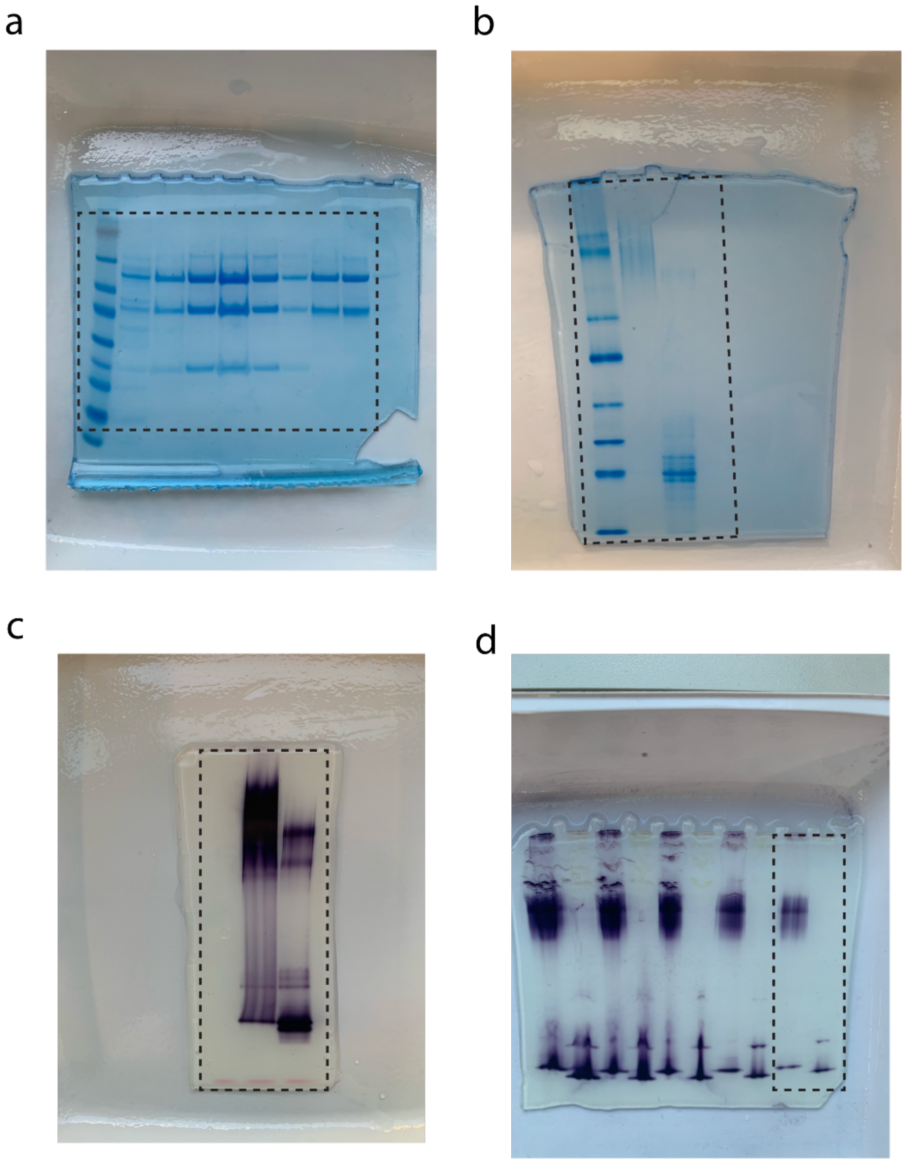

Supplementary Figure 1. Raw gel images from Huc purification. (a) Coomassie stained SDS-PAGE gel of Superose 6 SEC purified Huc shown in Extended Data Figure 1a. (b) Coomassie stained and (c) NBT activity stained native-PAGE gel of Huc oligomer and lower molecular weight species shown in Extended Data Figure 1c. (d) NBT activity stained native-PAGE gel of cell-lysates from *M. smegmatis* mc^{2}155 PRC1 HucS2×StrII wildtype and *ΔhucM* shown in Extended Data Figure 1e. Dotted lines show cropped region included in Extended Data Figure 1.

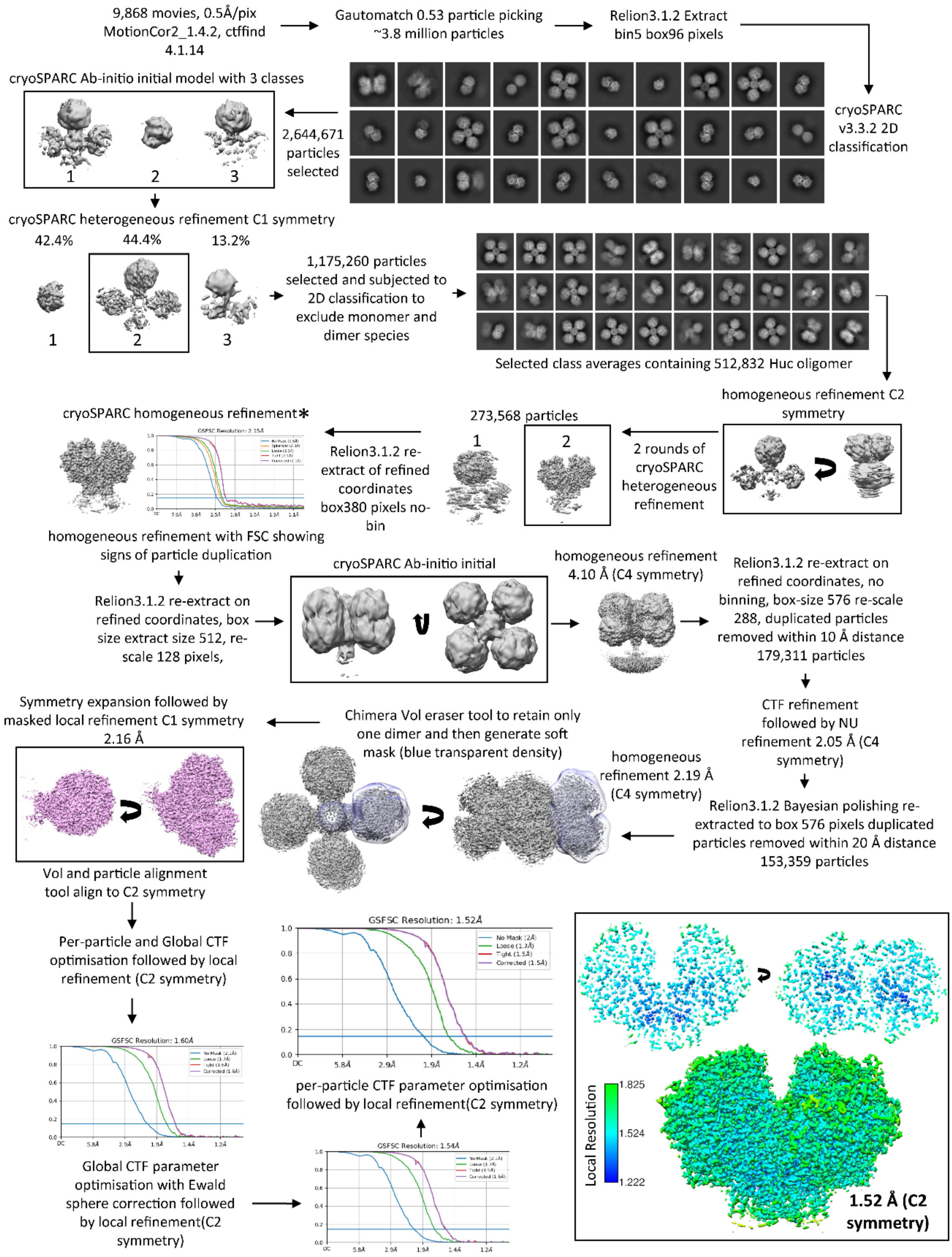

Supplementary Figure 2. Data processing workflow for the 1.52 Å Huc dimer reconstruction.

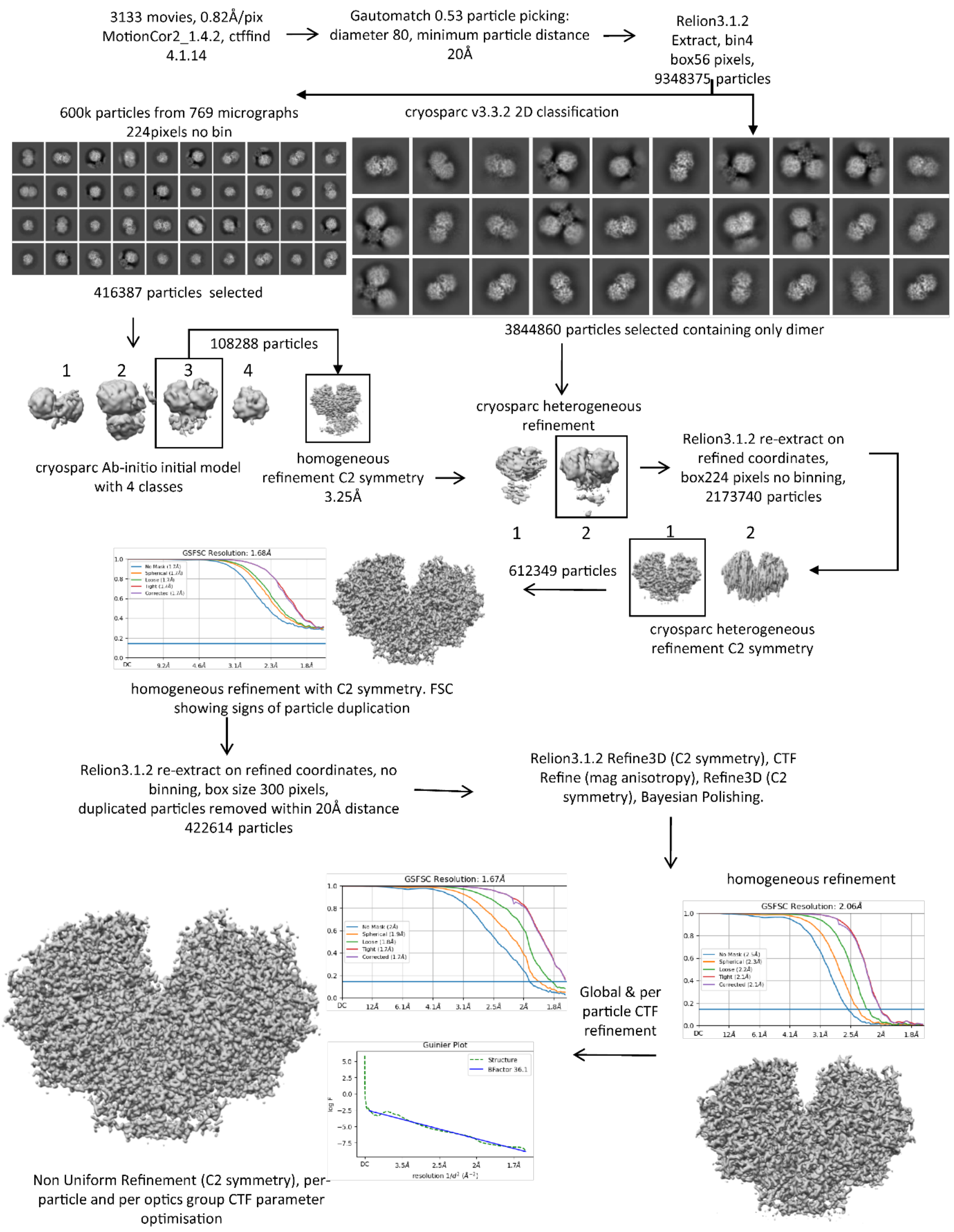

Supplementary Figure 3. Data processing workflow for the 1.67 Å Huc dimer reconstruction.

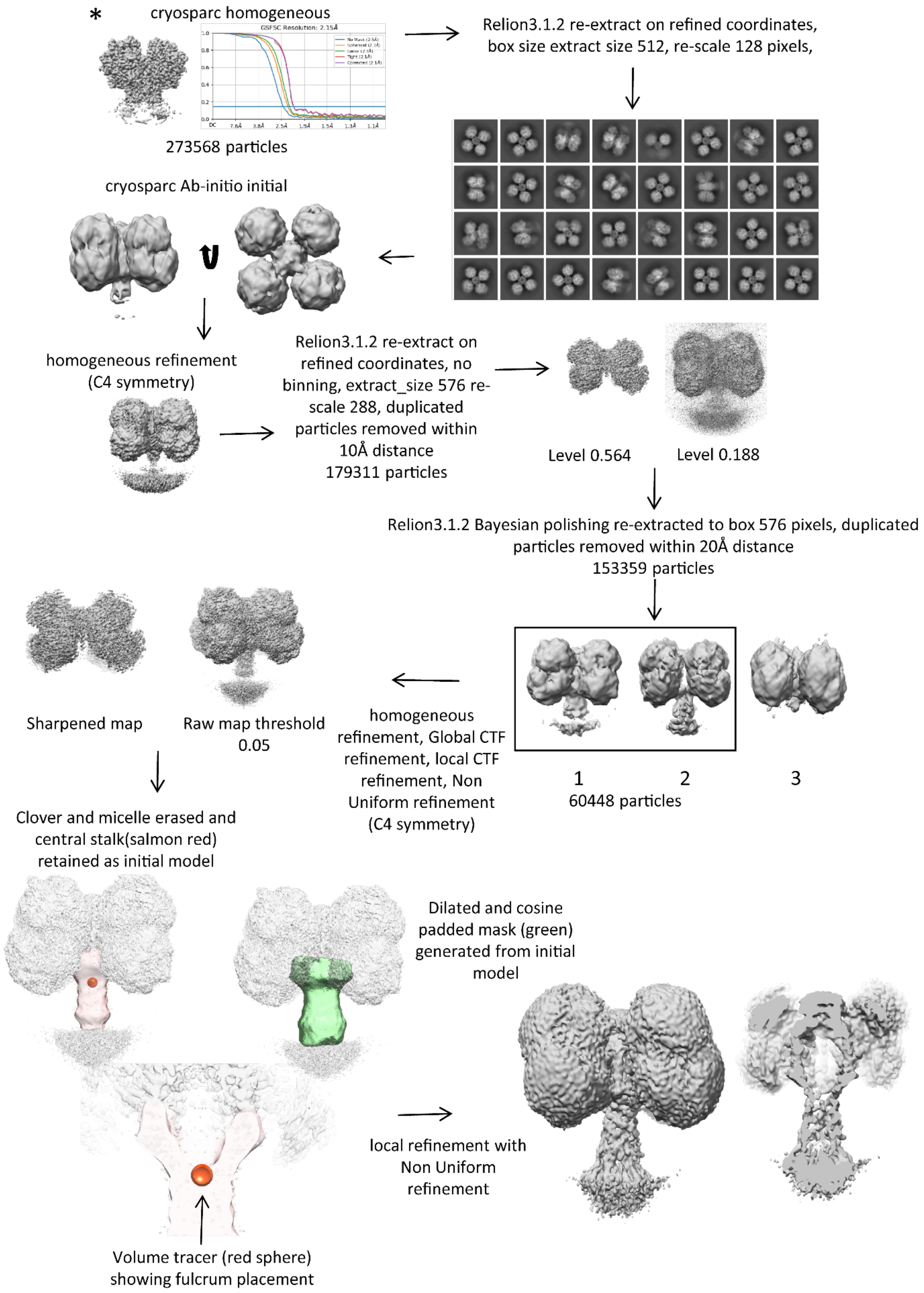

Supplementary Figure 4. Data processing workflow for Huc stalk reconstruction. * corresponds to particles from homogeneous refinement indicated in Supplemental Fig. 1.

a

b

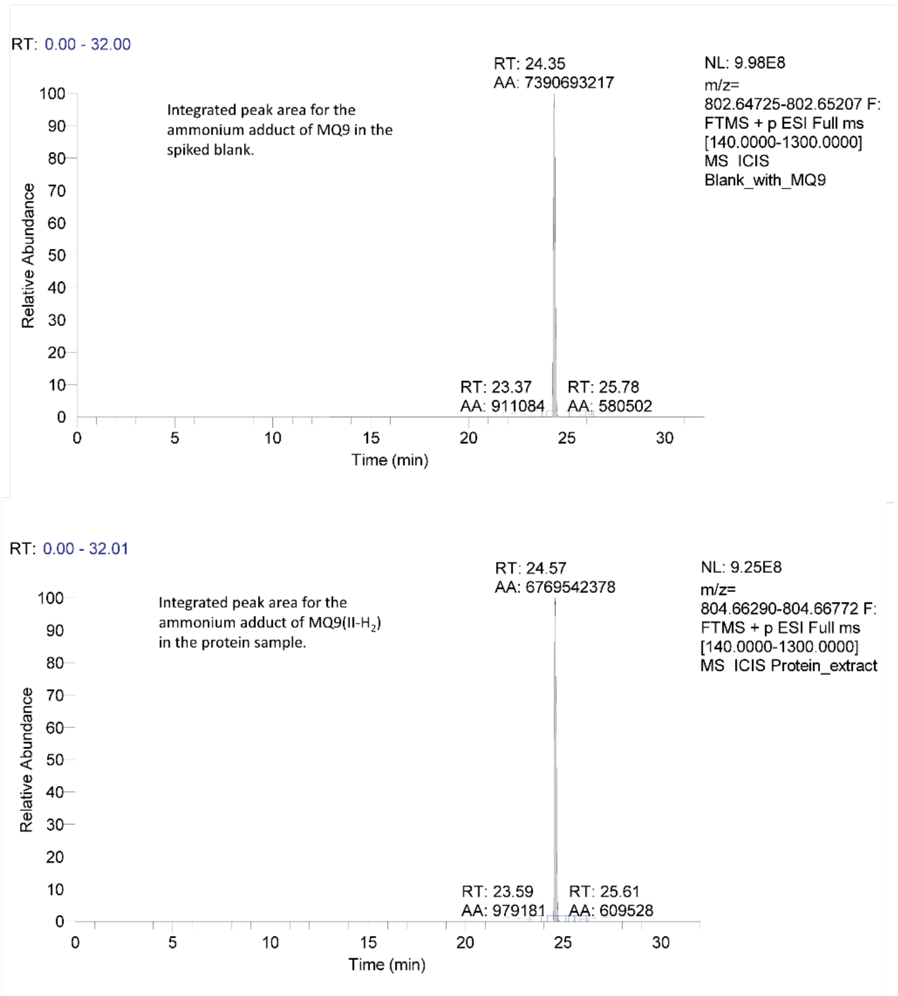

Supplementary Figure 5. Identification and quantitation of beta-dihydromenaquinone-9 (MQ9(II-H_2)) associated with Huc. (a) LC-MS trace of the 552 pmoles of the menaquinone 9 (MQ9) standard. (b) LC-MS trace of MQ9(II-H_2) extracted from 68.4 pmoles of purified Huc. Based on the MQ9 standard, this peak corresponds to 525.9 pmoles of MQ9(II-H_2), which with eight binding sites per Huc molecule, gives an occupancy of 96.1%.

Supplementary Table 1. Huc H_2 oxidation kinetic parameters

Acceptor Substrate	V_{max} (µmoles/min/mg)	K_m (nM)	K_{cat} (s^{-1})	K_{cat}/K_m ($M^{-1} s^{-1}$)
Menadione	4.09 (3.90-4.29)	129.0 (105.6-157.2)	7.05 (6.92-7.61)	5.47E+07
NBT	1.26 (1.12-1.43)	202.0 (128.4-313.4)	2.22 (1.99-2.54)	1.10E+07
Benzyl viologen	0.365 (0.332-0.402)	197.2 (134.2-286.7)	0.64 (0.59-71)	3.25E+06
O_2	1.08 (1.00-1.18)	114.4 (76.5-167.2)	2.04 (1.78-2.09)	1.79E+07
No Acceptor	0.457 (0.422-0.496)	275.4 (204.6-366.8)	0.81 (0.75-0.88)	2.94E+06

Bracketed numbers represent 95% CI (profile likelihood)

Supplementary Table 2. Caver-generated hydrogenase gas channel statistics

ID	Avg. Bottleneck radius (Å)	Max Bottleneck radius (Å)	Avg. Length (Å)	Avg throughput priority
Huc from *Mycobacterium smegmatis* (O_2 insensitive)				
1	**1.034**	1.03	27.304	0.25932
2	0.948	0.95	28.537	0.22188
3	0.957	0.96	30.589	0.19652
4	0.948	0.95	35.091	0.14496
Type 1h hydrogeanse from *Cupriavidus necator* H16 (PDB ID = 5AA5) (O_2 insensitive)				
1	**0.952**	0.95	28.005	0.31936
2	0.909	0.91	27.548	0.28061
3	0.885	0.89	40.237	0.15381
4	0.952	0.95	47.777	0.15043
[NiFe] hydrogenase from *Desulfovibrio gigas* (PDB ID = 2FRV) (O_2 sensitive)				
1	**1.518**	1.52	37.723	0.41316
2	1.052	1.05	28.558	0.31505
3	1.316	1.32	36.781	0.30486
4	1.444	1.44	51.77	0.26572
[NiFe] hydrogenase from *Desulufovibrio Vulgaris* Miyazaki F (PDB ID = 4U9H) (O_2 sensitive)				
1	**1.287**	1.29	33.591	0.36168
2	1.287	1.29	31.441	0.32027
3	1.21	1.21	38.573	0.28267
4	0.913	0.91	34.763	0.19519
E. coli Hydrogenase-2 (PDB ID=6EHQ) (O_2 sensitive)				
1	**1.305**	1.31	33.801	0.39878
2	1.43	1.43	33.432	0.34791
3	1.029	1.03	32.069	0.29154

4	1.081	1.08	29.847	0.27352

Oxygen tolerant [NiFe] hydrogenase from *Salmonella enterica* (PDB ID = 4C3O) (O_2 tolerant)

1	**1.441**	1.44	34.288	0.34255
2	1.163	1.16	37.374	0.28628
3	1.14	1.14	33.895	0.25205
4	1.047	1.05	30.572	0.25145

Membrane-bound respiratory [NiFe] hydrogenase from *Hydrogenovibrio marinus* (PDB ID = 3AYX) (O_2 tolerant)

1	**1.208**	1.21	36.227	0.32909
2	0.941	0.94	27.222	0.31974
3	0.912	0.91	33.1	0.21963
4	1.007	1.01	37.271	0.19823

O2-tolerant [NiFe]-hydrogenase from *Ralstonia eutropha* (PDB ID = 5MDK) (O_2 tolerant)

1	**1.251**	1.25	33.833	0.39858
2	0.984	0.98	28.691	0.28596
3	0.996	1	38.748	0.23665
4	1.251	1.25	52.308	0.20823

Supplementary Table 3. Comparison of Huc FTIR spectral states with previously characterised hydrogenases (adapted from Ash et al.[1]) (units = cm^{-1})

Hydrogenase	Group	Ni-A	Ni-B	Ni-SU	Ni-SI$_r$	Nia-SI	Ni$_a$-C	Ni$_a$-L$_I$	Ni$_a$-L$_{II}$	Ni$_a$-L$_{III}$	Ni$_a$-R$_I$	Ni$_a$-R$_{II}$	Ni$_a$-R$_{III}$
***M. smegmatis* Huc**	**2A**	**-**	**1950**	**-**	**-**	**1947**	**1963**	**1898**	**-**	**-**	**1937**	**-**	**-**
D. vulgaris Miyazaki F	1	1956	1955	1946	1922	1943	1961	-	1911	1890	1948	1932	1919
D. gigas	1	1947	1946	1950	1914	1934	1952	-	-	-	1940	1923	-
D. fructosovorans	1	1947	1946	1950	1913	1933	1951	-	-	-	1938	1922	-
A. vinosum MBH	1	1945	1943	1948	1910	1931	1951	1898	-	-	1936	1921	1913
T. roseopersicina MBH	1	-	1944	-	-	1930	1951	1899	-	-	-	1921	1915
R. eutropha MBH	1	-	1948	1943	1910	1936	1957	1899	-	-	1948	1926	1919
E. coli Hyd1	1	-	1943	-	-	1927	1949	1898	1877	1867	-	1922	1914
A. aeolicus MBH	1	-	1939	-	-	1927	1949	1862	1876	1900	-	1910	-
R. eutropha RH	2	-	1951	1957	1938	1942	1961	-	-	-	1949	1934	1918
P. furiosus SH1	3	-	1960	-	1931	1950	1967	1917	1922	-	1954	1940	1931
R. eutropha SH	3	-	1957	-	1946	-	1961	-	-	-	1958	1922	1913
Synechocystis SH	3	-	1957	-	-	1947	1968	-	-	-	1955	-	-

Supplementary Table 4. Bacterial strains and plasmids used in this study

**Primer Name**	**Sequence 5'-3'**	**Purpose**
Huc Strep tag Forward	GGCGGCGGCGAAAACCTGTAT	Screening of strep tag
Huc chromosome Forward	CGGCGATCGAGAGGAGACCC	Screening of strep tag
Huc chromosome Reverse	CTTCTTTGGACCACTGCGGTGC	Screening of strep tag
HucM Flank 1 Forward	GCATGACTAGTCGATGTAGTCGACGGCCTGCTG	Amplification of HucM flanks for deletion construct
HucM Flank 1 Reverse	GCATGCATATGTCCATCCTCCCGCTCGGAGTTTCGG	Amplification of HucM flanks for deletion construct
HucM Flank 2 Forward	GCATGCATATGTCGAAGCGAAAACCTTTACAGTCCAGC	Amplification of HucM flanks for deletion construct
HucM Flank 2 Reverse	GCATGACTAGTCGCCGCCAGACCCATGTACGTC	Amplification of HucM flanks for deletion construct
**Strain or plasmid**	**Relevent genotype and description**	**Reference or source**
Escherichia coli K12 (DH5α)	Standard laboratory *E. coli* strain for plasmid propogation and maintenance	-
Mycobacterium smegmatis mc²155 gylR(Leu154->Frameshift) (*hhyS* Inactivated by N-term Strep-tagging)	Isogenic strain to WT except with frameshift mutation (Leu154) to gylR gene	Cordero[2]
Mycobacterium smegmatis mc²155 gylR(Leu154->Frameshift) (*hhyS* Inactivated by N-term Strep-tagging) HucS-2xStrep	Isogenic strain to WT except with frameshift mutation (Leu154) to gylR gene and 2xStrep tag on HucS N terminus	This study
Mycobacterium smegmatis mc²155 gylR(Leu154->Frameshift) (*hhyS* Inactivated by N-term Strep-tagging) HucS-2xStrep ΔHucM	Isogenic strain to WT except with frameshift mutation (Leu154) to gylR gene, 2xStrep tag on HucS N terminus and deletion of HucM gene	This study

Supplementary Note 1. Analysis of Huc by EPR spectroscopy

We used EPR spectroscopy to assess the configuration of the HucS iron-sulfur clusters. At 30 K, the EPR spectrum of the "as-isolated" Huc was dominated by a featureless, isotropic signal centered at *g* = 2.03. This signal lacks the sharp, low-field feature and anisotropic lineshape that is typical for an isolated, oxidized (S = ½) $[3Fe\text{-}4S]^{+}$ cluster[3,4], likely due to spin-spin interactions between the clusters that broaden the spectral features (Fig 3c)[5-8]. At a lower temperature (7 K) (Extended Data Fig 5b), this *g* ~2 region displayed a slightly rhombic signal, which may be attributed to the $[3Fe\text{-}4S]^{+}$ clusters interacting with the nearby [Ni-Fe] center. Flushing Huc with H_2 resulted in an essentially EPR silent sample both at 30 K and 7 K, as expected due to the reduction of the FeS clusters to their EPR-inactive $[3Fe\text{-}4S]^{0}$ form (Fig. 3c). Importantly, no broad rhombic signals attributable to $[4Fe\text{-}4S]^{+}$ clusters were discernible in spectra recorded of the H_2-reduced Huc (Extended Data Fig 5b)[5,6]. These results are in good agreement with the structural data indicating Huc exclusively binds high potential $[3Fe\text{-}4S]^{1+/0}$ clusters rather than low potential $[4Fe\text{-}4S]^{2+/1+}$ clusters.

Upon close inspection of the low-field side of the EPR spectrum, signals characteristic of the [NiFe] active site are observed (Extended Data Fig 5c). In the oxidized state, a partially rhombic signal in good agreement with the canonical Ni-B state of the [NiFe] site is clearly visible in the high temperature (40 K) spectrum. Most features in the spectrum can be simulated with g-values of 2.36, 2.21, and a third turning point that is unresolved due to overlap with the $[3Fe\text{-}4S]^{+}$ cluster signal (Extended Data Fig 5c). Spectra collected at lower temperatures reveal significantly more complex signals with substantially increased spectral breadth and anisotropy (Extended Data Fig 5c). These spectral features most likely arise from strong spin-spin interactions between the active site and the $[3Fe\text{-}4S]^{+}$ clusters, albeit an alternative electronic structure at the active site (e.g., distinct protonation state, H-bonding environment, electron density at the Ni center) cannot be completely excluded. In the H_2-reduced state, the spectral breadth is reduced, as is typically seen for the EPR-active Ni-C state (Extended Data Fig 5c). Again, a complex spectrum is observed, suggesting the presence of multiple species with slight electronic distinctions or splitting due to interactions with the clusters, which, even in the EPR-silent reduced state, likely remain paramagnetic. Work towards elucidating the active-site electronic structure through EPR spectroscopy is underway but beyond the scope of this report.

Supplementary Note 2. Putative mechanisms of O_2 tolerance and insensitivity

In previously studied respiratory O_2-tolerant hydrogenases, it is proposed that O_2 binds to the [NiFe] active site and is rapidly reduced by four electrons to the innocuous oxidation state of water. Three of these electrons derive from normal redox transitions of the medial and proximal iron-sulfur clusters of the small subunit along with the active site itself[9-12]. The fourth electron originates from an unusual

high-potential redox transition of the unusual [4Fe-3S] proximal cluster. By removing the need for long-distance electron transfer from the distal cluster, there is no kinetic hurdle for the complete reduction of O_2 to hydroxide[13], where, upon protonation, it can be easily released from the [NiFe] cluster[11,12,14]. A similar mechanism is proposed for O_2 inactivation in O_2-sensitive hydrogenases; however, because the fourth electron transfer must derive from the distal cluster, the rate of complete reduction is slowed, enabling competing degradative pathways.

As Huc exclusively contains [3Fe-4S] clusters, it is unable to fully reduce O_2 in this way and must resist O_2 inactivation by a distinct mechanism. Oxidation of the enzyme by O_2 to give this Ni-B-like state may occur via one of two plausible mechanisms: In the direct pathway, an O_2 molecule that occasionally reaches the active site can be reduced with three electrons from the [3Fe-4S]^0 clusters along with one from the Ni centre to release water, yielding a hydroxide-bound, oxidized nickel. In the indirect pathway, which is in line with previous studies showing the O_2-independent formation of Ni-B and Ni-B-like states[15,16], outer-sphere oxidation of the [3Fe-4S] clusters by O_2 may induce oxidation of the [NiFe] centre through intramolecular electron transfer. The resultant electron-deficient Ni ion would be prone to coordinate water with concomitant deprotonation to yield a hydroxide ligand. In either case, analogous to observations in the O_2-tolerant membrane-bound hydrogenases, Ni-B formation may be protective, preventing further reactivity with O_2, while the oxidized redox chain would resist further reaction with O_2. Protonation of the hydroxide ligand to a labile water would enable facile displacement by an H_2 molecule through a standard ligand substitution reaction[17]. The kinetics observed by FTIR spectroscopy support the indirect pathway. When reduced Huc is exposed to an atmosphere containing 20% O_2, the Ni-SI state is first populated, at the expense of the reduced Ni-C and Ni-R states, followed by the appearance of the hydroxide-bound Ni-B state (Extended Data Fig. 8e,f). This suggests sequential one-electron oxidation processes, as would be expected for the indirect pathway. On the other hand, FTIR spectra indicate the direct pathway is operative in the O_2-sensitive Hyd2 enzyme from *E. coli* (Extended Data Fig. 8f)[18], where exposure to O_2 results in the near-immediate loss of all catalytically relevant states (Ni-C, Ni-R, Ni-SI) with concomitant formation of the multi-electron-oxidized “ready” inactive Ni-B and “unready” inactive Ni-A states.

Supplementary Note 3. MD simulations of H_2 and O_2 diffusion towards the Huc active site

To test the hypothesis that narrowing of the hydrophobic tunnels that provide access to the Huc active site contributes to the O_2 insensitivity of the enzyme, we performed molecular dynamics simulations

on the HucSL dimer in the presence of an excess of either H_2 or O_2. Simulations were run for 50 nanoseconds (ns) and repeated three times. Huc was stable and retained its original secondary structure throughout the simulations. H_2 penetrated the Huc gas channels, with the molecule reaching within 8 Å of the active site within the first 10 ns of the simulations (Extended Data Fig 6b). O_2 entered the gas channels more slowly but did reach within 8 Å of the active site in the second half of the simulations (Extended Data Fig 6b). By the end of the simulations, comparatively similar numbers of H_2 and O_2 molecules reached within 12-14 Å of the active site (approximately half the length of the Huc gas channel) (Supplementary Table 2). However, a much smaller number of O_2 molecules reached within 8-10 Å of the active site compared to H_2 (Extended Data Fig 6b). This indicates that bottlenecks along the length of the tunnel selectively limit O_2 access to the interior of Huc, which is consistent with the varying diameter of the gas channels calculated using the CAVER3 code (Fig. 3f)[19]. As discussed in the main text, an additional bottleneck in the Huc gas channel prevented O_2 from reaching a proximity of closer than 5 Å to the Huc catalytic cluster in our simulations (Extended Data Fig 6c,d). Conversely, in a number of simulation frames, H_2 molecules are observed within 3 Å of the catalytic cluster and, in several instances, in a location analogous to the ligand-bound state of the cluster (Extended Data Fig 6e,f). This indicates that the ultimate point selection against O_2 is a bottleneck after the convergence of the three gas tunnels immediately preceding the active site entrance. Interestingly, the conformation of amino acids in this region appears responsive to whether Huc was simulated in the presence of H_2 or O_2. In the simulations in the presence of O_2, arginine 443 from HucL largely adopted a conformation where it formed a close hydrogen bond with serine 104 from HucL (Extended Data Fig 6h,i). This state was never fully realized in the simulations with H_2 and may be indicative of long-range structural changes that prime Huc for O_2 exclusion from the catalytic site (Extended Data Fig. 6g,i).

To assess the role of the bottleneck immediately preceding the Huc active site in excluding O_2, we simulated Huc models in which key residues that form it (glutamate 15, isoleucine 64 and leucine 122) were mutated to the less bulky sidechain alanine, either in pairs (E15A + I64A; I64A + L122A) or a triple mutant (E15A + I64A + L122A). Simulations were run on these mutants and wildtype Huc (WT) for 100 ns and repeated three times. As for the previous simulations, for WT and the I64A + L122A, O_2 was unable to reach closer than 4.7 Å to the catalytic cluster (Extended Data Fig. 7a,b). However, for the E15A + I64A and E15A + I64A + L122A mutants, O_2 molecules reached within bonding distance of the catalytic cluster during the simulations (Extended Data Fig. 7a,b). In both mutants, the O_2 molecules were able to access a region below the active site in close proximity to the $Fe(CN)_2CO$ cofactor, while in the E64A + I64A mutant O_2 molecules reached the active site nickel ion (Extended Data Fig. 7c). Based on these simulations we hypothesize that both these mutants would be sensitive to O_2

inhibition. However, further experimental evidence is required to confirm the role of the pre-active site bottleneck in the O_2 insensitivity of Huc.

Supplementary Note 4. Analysis of Huc redox states by aerosol-controlled ATR-FTIR spectroscopy

To understand the redox states adopted by the Huc [NiFe] cluster, we recorded its FTIR spectra sequentially in ambient air, H_2, and N_2. Huc prepared under ambient conditions (78.09% N_2, 20.95% O_2, 0.000053% H_2) was analysed under 100% N_2. Under these conditions, the FTIR spectra were assigned to the oxidized Ni-B state, which possesses an OH^- ligand bridging the catalytic Ni and Fe ions (Fig. 3d, 3e), since the Cryo-EM structure obtained under similar conditions shows only density for one oxygen atom (Fig. 3a), which renders the formation of the Ni-A state with a peroxo ligand (OOH^-) unlikely (Extended Data Fig. 8a,b)[20-22]. This indicates that the Huc [NiFe] cluster is fully oxidized by the O_2 present in ambient air. When the atmosphere in the cell was exchanged for $^2H_2$, the Ni-B signal diminished, and spectra consistent with a high proportion of the catalytic Ni-R and Ni-C state and a lesser amount of Ni-SI state were observed (Extended Data Fig. 8a,b,g,h). This indicates that the oxidized Ni-B state of Huc can rapidly enter the H_2 oxidation cycle when H_2 becomes available. The subsequent exchange of the cell atmosphere for N_2 led predominantly to population of the Ni-SI state, a reduced, non-hydrogen bound state possibly through auto-oxidation by Huc-associated menaquinone (Extended Data Fig. 8a,b). A further exchange of the atmosphere with H_2 led to a similar spectrum to that observed for $^2H_2$ (Extended Data Fig. 8a,b). Finally, when the atmosphere was exchanged for air, the proportion of the Ni-B state increased at the expense of the reduced states, confirming the role of O_2 in oxidizing the enzyme to this state (Extended Data Fig. 8a,b). Taken together, these data indicate that, like O_2-tolerant membrane-bound hydrogenases, the Huc [NiFe] cluster oxidises to the hydroxyl-bound Ni-B state in the presence of O_2[20,23,24].

To better understand the kinetics of the redox state transitions of the Huc [NiFe] cluster, we collected time-resolved FTIR spectra after transferring Huc oxidised in ambient air into a 100% H_2 atmosphere. Over a timescale of minutes, the Ni-R and Ni-C states were populated at the expense of Ni-B with a near-equilibrium reached by 350 seconds (Extended Data Fig. 8c). In contrast, when reduced Huc populating predominantly Ni-R and Ni-SI states was transferred from 100% N_2 into 100% H_2, it rapidly populated the Ni-R state at the expense of Ni-SI, equilibrium was achieved within 50 seconds (Extended Data Fig. 8d). These data indicate that Huc in the hydroxide bound Ni-B state reacts more slowly with H_2 than in the reduced non-hydrogen Ni-SI bound state. This is consistent with the [NiFe] bound hydroxyl in the Ni-B state needing to be displaced by H_2 for the catalytic cycle to proceed. To assess the rate response of the Huc [NiFe] cluster when exposed to O_2, we collected time-resolved

FTIR spectra after transferring Huc in a mixed Ni-SI, Ni-R, and Ni-C state from 100% N_2 into 20%:80% N_2 to O_2. Initially, the Ni-SI state was further populated at the expense of Ni-R and Ni-C, before the Ni-SI state was replaced by Ni-B over a timescale of minutes (Extended Data Fig. 8e). These data suggest that the hydrogen-bound Ni-R and Ni-C states must first convert to the empty reduced Ni-SI state before the hydroxide-bound oxidised Ni-B state is formed (Extended Data Fig. 8g,h). This is in contrast to the oxidation profile observed for the oxygen-sensitive hydrogenase Hyd2 from *E. coli* which was previously shown to rapidly adopt Ni-A and Ni-B states at the expense of all other states when exposed to only 1% O_2 (Extended Data Fig. 8f)[18]. These data indicated that the Huc [NiFe] cluster is oxidised much more slowly than oxygen-sensitive hydrogenases, likely due to the restricted access of O_2 to its active site and potentially because of the altered properties of its redox chain.

References for supplementary notes

1 Ash, P. A., Hidalgo, R. & Vincent, K. A. Proton transfer in the catalytic cycle of [NiFe] hydrogenases: insight from vibrational spectroscopy. *ACS catalysis* **7**, 2471-2485 (2017).

2 Cordero, P. R. Cellular and molecular basis of atmospheric hydrogen and carbon monoxide oxidation in mycobacteria. *PhD Thesis (Monash University)* (2020).

3 Knaff, D., Hirasawa, M., Ameyibor, E., Fu, W. & Johnson, M. Spectroscopic evidence for a [3Fe-4S] cluster in spinach glutamate synthase. *Journal of Biological Chemistry* **266**, 15080-15084 (1991).

4 Lewis, L. C. & Shafaat, H. S. Reversible Electron Transfer and Substrate Binding Support [NiFe3S4] Ferredoxin as a Protein-Based Model for [NiFe] Carbon Monoxide Dehydrogenase. *Inorganic Chemistry* **60**, 13869-13875 (2021).

5 Rousset, M. *et al.* [3Fe-4S] to [4Fe-4S] cluster conversion in *Desulfovibrio fructosovorans* [NiFe] hydrogenase by site-directed mutagenesis. *Proceedings of the National Academy of Sciences* **95**, 11625-11630 (1998).

6 Land, H. *et al.* Characterization of a putative sensory [FeFe]-hydrogenase provides new insight into the role of the active site architecture. *Chemical science* **11**, 12789-12801 (2020).

7 Cox, N. *et al.* A tyrosyl− dimanganese coupled spin system is the native metalloradical cofactor of the R2F subunit of the ribonucleotide reductase of *Corynebacterium ammoniagenes*. *Journal of the American Chemical Society* **132**, 11197-11213 (2010).

8 Dole, F. *et al.* Spin− Spin Interactions between the Ni Site and the [4Fe-4S] Centers as a Probe of Light-Induced Structural Changes in Active *Desulfovibrio gigas* Hydrogenase. *Biochemistry* **35**, 16399-16406 (1996).

9 Wulff, P., Day, C. C., Sargent, F. & Armstrong, F. A. How oxygen reacts with oxygen-tolerant respiratory [NiFe]-hydrogenases. *Proceedings of the National Academy of Sciences* **111**, 6606-6611 (2014).

10 Pandelia, M.-E. *et al.* Characterization of a unique [FeS] cluster in the electron transfer chain of the oxygen tolerant [NiFe] hydrogenase from *Aquifex aeolicus*. *Proceedings of the National Academy of Sciences* **108**, 6097-6102 (2011).

11 Fritsch, J. *et al.* The crystal structure of an oxygen-tolerant hydrogenase uncovers a novel iron-sulphur centre. *Nature* **479**, 249-252 (2011).

12 Shomura, Y., Yoon, K.-S., Nishihara, H. & Higuchi, Y. Structural basis for a [4Fe-3S] cluster in the oxygen-tolerant membrane-bound [NiFe]-hydrogenase. *Nature* **479**, 253-256 (2011).

13 Parkin, A. & Sargent, F. The hows and whys of aerobic H_2 metabolism. *Current opinion in chemical biology* **16**, 26-34 (2012).
14 Goris, T. *et al.* A unique iron-sulfur cluster is crucial for oxygen tolerance of a [NiFe]-hydrogenase. *Nature chemical biology* **7**, 310-318 (2011).
15 Abou Hamdan, A. *et al.* O_2-independent formation of the inactive states of NiFe hydrogenase. *Nature chemical biology* **9**, 15-17 (2013).
16 Fourmond, V., Infossi, P., Giudici-Orticoni, M.-T. r. s., Bertrand, P. & Léger, C. "Two-step" chronoamperometric method for studying the anaerobic inactivation of an oxygen tolerant NiFe hydrogenase. *Journal of the American Chemical Society* **132**, 4848-4857 (2010).
17 Kubas, G. J. Fundamentals of H_2 binding and reactivity on transition metals underlying hydrogenase function and H_2 production and storage. *Chemical reviews* **107**, 4152-4205 (2007).
18 Senger, M., Laun, K., Soboh, B. & Stripp, S. T. Infrared Characterization of the Bidirectional Oxygen-Sensitive [NiFe]-Hydrogenase from *E. coli. Catalysts* **8**, 530 (2018).
19 Chovancova, E. *et al.* CAVER 3.0: a tool for the analysis of transport pathways in dynamic protein structures. PLOS Computational Biology 8(10): e1002708. (2012).
20 Lubitz, W., Ogata, H., Rudiger, O. & Reijerse, E. Hydrogenases. *Chemical reviews* **114**, 4081-4148 (2014).
21 van Gastel, M. *et al.* A single-crystal ENDOR and density functional theory study of the oxidized states of the [NiFe] hydrogenase from *Desulfovibrio vulgaris* Miyazaki F. *JBIC Journal of Biological Inorganic Chemistry* **11**, 41-51 (2006).
22 Ogata, H. *et al.* Activation process of [NiFe] hydrogenase elucidated by high-resolution X-ray analyses: conversion of the ready to the unready state. *Structure* **13**, 1635-1642 (2005).
23 Evans, R. M. *et al.* Mechanistic exploitation of a self-repairing, blocked proton transfer pathway in an O_2-tolerant [NiFe]-Hydrogenase. *Journal of the American Chemical Society* **140**, 10208-10220 (2018).
24 Stein, M. & Lubitz, W. DFT calculations of the electronic structure of the paramagnetic states Ni-A, Ni-B and Ni-C of [NiFe] hydrogenase. *Physical Chemistry Chemical Physics* **3**, 2668-2675 (2001).
